# Controlled, blinded force platform analysis of the effect of intraarticular injection of autologous adipose-derived mesenchymal stem cells associated to PRGF-Endoret in osteoarthritic dogs

**DOI:** 10.1186/1746-6148-9-131

**Published:** 2013-07-02

**Authors:** Jose M Vilar, Manuel Morales, Angelo Santana, Giuseppe Spinella, Mónica Rubio, Belen Cuervo, Ramón Cugat, Jose M Carrillo

**Affiliations:** 1 Department of Animal Pathology, Faculty of Veterinary Medicine, Universidad de Las Palmas de Gran Canaria, Trasmontaña S/N, Arucas 35413 Las Palmas, Spain; 2 Department of Veterinary Science, University Alma Mater of Bologna, via Tolara di Sopra 50, Ozzano dell’Emilia (BO), Italy; 3 Departamento de Medicina y Cirugía Animal, Universidad CEU Cardenal Herrera, C/ Tirant lo Blanc, 7, 46117 Alfara del Patriarca Valencia, Spain; 4 Fundación García Cugat, Madrazo 43, 08006 Barcelona, Spain; 5 Instituto de Ciencias Biomédicas Universidad CEU Cardenal Herrera, C/del pozo s/n, 46115 Alfara del Patriarca Valencia, Spain; 6 Artroscopia GC. Hospital Quirón Barcelona, Plaza Alfonso Comí, 12, Barcelona, Spain

**Keywords:** Osteoarthrosis, Hip, Adipose-derived mesenchymal stem cells, Force platform, PRGF

## Abstract

**Background:**

Adipose-derived mesenchymal stem cell (ADMSC) therapy in regenerative medicine is a rapidly growing area of research and is currently also being used to treat osteoarthritis (OA). Force platform analysis has been consistently used to verify the efficacy of different therapeutic strategies for the treatment of OA in dogs, but never with AD-MSC.

The aim of this study was to use a force platform to measure the efficacy of intraarticular ADMSC administration for limb function improvement in dogs with severe OA.

**Results:**

Eight lame dogs with severe hip OA and a control group of 5 sound dogs were used for this study. Results were statistically analyzed to detect a significant increase in peak vertical force (PVF) and vertical impulse (VI) in treated dogs. Mean values of PVF and VI were significantly improved after treatment of the OA groups, reaching 53.02% and 14.84% of body weight, respectively, at day 180, compared with only 43.56% and 12.16% at day 0.

**Conclusion:**

This study objectively demonstrated that intraarticular ADMSC therapy resulted in reduced lameness due to OA.

## Background

Osteoarthritis (OA) is a common disorder in veterinary medicine, and clinicians are increasingly encountering this condition [1,2]. However, restoration of the diseased articular cartilage in patients with OA is still a challenge for researchers and clinicians. Currently, several experimental strategies have investigated whether mesenchymal stem cells (MSCs), instead of chondrocytes, can be used for the regeneration and maintenance of articular cartilage in OA [3].

Autologous stem cell therapy in the field of regenerative veterinary medicine involves harvesting tissue,such as fat or bone marrow [4], from the patient, isolating the stem and regenerative cells, and administering the cells back to the patient [5]. The field of adipose-derived MSC therapy (ADMSC) is a rapidly growing area of research, and it has been shown that stem cells have an affinity for damaged joint tissue; recent in vivo studies have confirmed that stem cells have the ability to localize and participate in the repair of damaged joint structures, including cruciate ligaments, menisci, and cartilage lesions [6]. For these reasons, stem cell therapy is now being used to treat OA.

Previous studies evaluating OA therapy in dogs suggest that non-steroidal anti-inflammatory drugs do not often provide complete pain relief [7], perhaps because they rely on a single target receptor or pathway for their action. In contrast to drug therapy, cellular therapies such as ADMSCs play a trophic function by recruiting endogenous cells to the injured site. Studies and anecdotal clinical experience demonstrate that autologous ADMSC therapy is of clinical benefit in horses and dogs with orthopedic conditions [8-12].

Recent investigations have shown that growth factors contained in platelet-rich plasma (PRGF) act as vehicles and even potentiators of the effect of MSCs [13,14]. The purpose of this study was to use force platform kinetic analysis to evaluate the effect of a single intraarticular injection of ADMSCs in 8 dogs with OA of hip joints by measuring peak vertical force (PVF) and vertical impulse (VI), which represent maximal weight bearing and distribution of forces through time, respectively.

## Results

The body weight of enrolled dogs ranged from 41 to 53 kg (mean ± SD = 47.1 ± 3.7 kg), and ages were 4 to 8 years (5.2± 1.7 years). The mean (± SD) value for walking velocity of both sound (control) and diseased groups of dogs was 1.6 ± 0.5 m/s. No significant difference in walking velocity existed between dogs (*P*= 0.06). PVF and VI mean values are summarized in Table 1.

**Table 1:** Mean and standard deviation of PVF and VI in % dog weight (N/N and N.s/N, respectively) applied on the diseased leg

**Day**	**0**	**30**	**90**	**180**
PVF ML	43.56 ± 0.73	49.21 ± 2.1	49.05 ± 1.51	53.2 ± 4.43
PVF LL	55.98 ± 0.62	53.96 ± 1.46	52.67 ± 1.91	51.53 ± 2.79
PVF S	47.40 ±1.43	47.74 ± 1.34	47.95 ±1.35	47.94 ± 1.65
VI ML	12.16 ± 0.72	13.75 ±1.17	13.71 ± 1.1	14.84 ± 1.29
VI LL	16.09 ± 1.01	15.51 ± 1.28	15.12 ± 1.45	14.83 ± 1.54
VI S	14.51 ± 0.47	14.62 ± 0.41	14.67 ± 0.42	14.67 ± 0.51

PVF ML: peak vertical force in the more-lame limbs. PVF LL: peak vertical force in the less-lame limbs. VI ML: vertical impulse in the more-lame limbs VI LL: vertical impulse in the less-lame limbs. PVF S: peak vertical force in the control group. VI S: vertical impulse in the control group.

Data are shown for each day of observation.

### Analysis of PVF

More-lame limbs analysis showed that differences in % PVF between D0 and D30 were significant (p-value< 0.001). Between the other periods, differences were of no significance (p-value > 0.349).

Compared with the control group, % PVF at D0 is significantly less (p-value< 0.001). In comparison, beyond this time differences became non significant (p-value >0.499).

Less-lame limbs analysis showed non significant differences till D30 (p value = 0.058); beyond D90, differences were significant (p-value<0.001).

**Figure 1:**
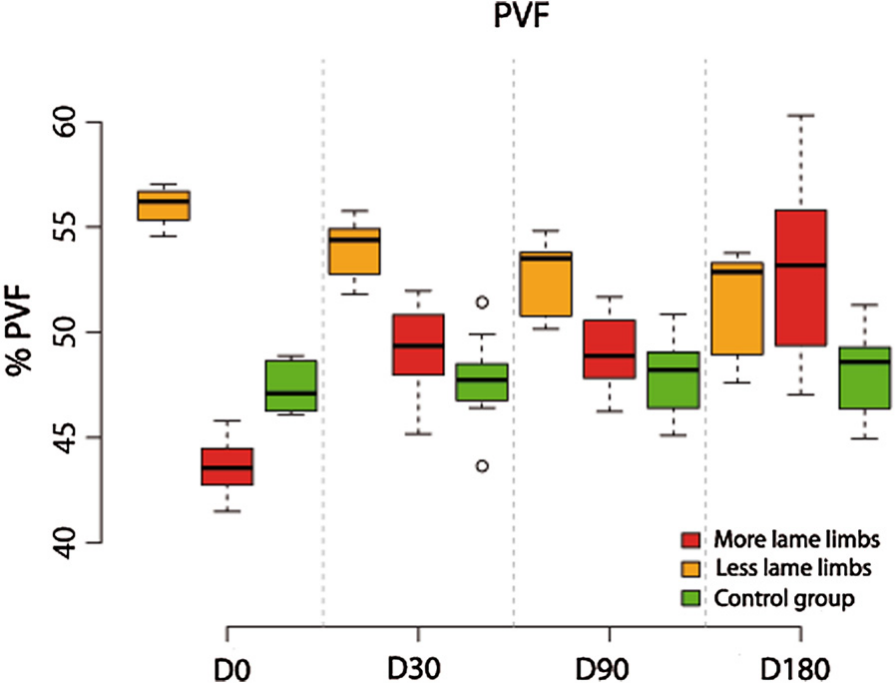
Evolution of PVF in lame group dogs after treatment at the 6-month follow-up period.

Compared with control group, % PVF at D0 is significantly greater at D0 (p-value < 0.001), D30 (p-value =0.03) and D90 (p-value=0.015). At D180 this difference was not significant (p-value =0.14) (Figure 1).

### Analysis of VI

More-lame limbs analysis showed that differences in % VI between D0 and D30 were significant (p-value< 0.001). With the other periods, differences were not significant (p-value > 0.05).

Compared with the control group, % VI at D0 is significantly less (p-val = 0.024). In comparison, beyond this time differences became not significant (p-value > 0.84.

Less-lame limbs analysis showed no significant differences at any control period (p value > 0.462).

**Figure 2:**
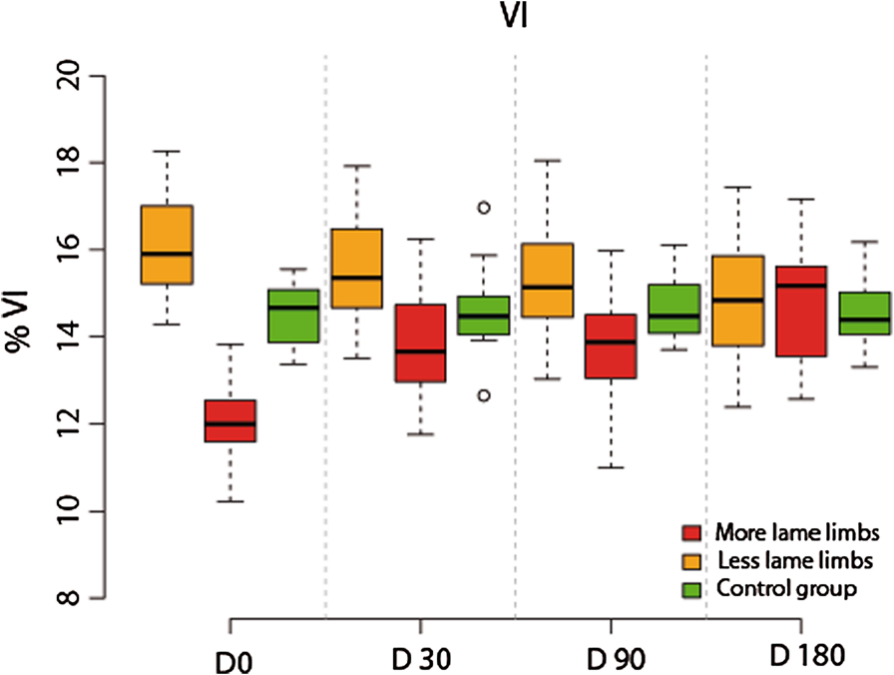
Evolution of VI in lame group dogs after treatment at the 6-month follow-up period.

Compared with the control group, no significant differences were also found (p-values > 0.876) (Figure 2).

### Association between more and less-lame limbs

The fitted linear mixed model shows a negative association between % PVF in less-lame limb and more-lame limb (β_1_<0, p-value=0.010). In % VI there is no association between VI in less and more -lame limb (β_1_=0, p-val=0.61).

The validity of the model fit was assessed by testing normality and homoscedasticity of the residuals. Both assumptions could be accepted: the Shapiro-Wilk test for normality and Levene’s test for homoscedasticity were not significant (P = 0.67 and P = 0.995, respectively).

## Discussion

In this trial, the effect of MSCs in lame OA dogs was investigated and quantified by means of an objective evaluation with a force platform. The ground reaction forces-related aspects of the gait, such as the PVF and VI, which represent maximal weight bearing and distribution of forces through time, respectively, measured the clinical impact of MSC treatment on the function of the limb during the stance phase of walking.

The absence of a direct relationship between radiographic evidence of OA and force platform findings is well known; in any case, in our study, diseased dogs were selected both on the basis of the presence or absence of radiographic evidence of severe OA (D-E degrees of hip dysplasia) and for evident lameness objectively determined by platform gait analysis [15].

Voss et al. [16] and Evans et al. [17] reported that force platform gait analysis at trot was much more sensitive than at walk for low-grade hindlimb lameness, but not for severe lameness. In our case, lameness of the OA dogs group was evident by direct observation, even at walk.

Although each dog had bilateral lameness, we believe that confident data could only be obtained from the more-lame limbs (lesser PVF), in order to limit a possible bias caused by inconsistent weight redistribution to the less affected contra-lateral hindlimb. In fact, mean values showed how initially less-lame limbs seemed to be “better” than limbs from the control group.

We observed a substantial improvement both in PVF and VI values through the period of evaluation, with an indicative gaining of limb function. Evolution of VI seems to be something controversial since some authors suggest that recordings of improvement in VI may suffer from a delay; their dogs tended to decrease their stance time in comparison with baseline values after non-steroidal anti-inflammatory drug treatment because they were able to improve their performance. However, other authors affirm that stance time did not change or increase when limb function improved [18-20]. In our study we observed independent evolution of both PVF and VI values, we hypothesize that this fact occurs because PVF only depends on the force exerted by the limbs, whilst VI reflects the evolution of the force during the whole support phase. For this reason VI could vary when one or both variables change (force and support time).

In 1999 Budsberg et al. compared the efficacy of a treatment(Etodolac) with a placebo control group and demonstrated a decrease in lameness in the treatment group using force platform analysis. In contrast, the degree of lameness in the dogs receiving the placebo deteriorated during the study period, making the control group unable to provide fixed reference data [21]. Based on their results, we designed the experimental study using a control group of sound dogs that were able to provide fixed reference data.

Different adipose tissue donor sites have been found in revised literature: retroperitoneal adipose tissue [22], lateral thoracic area [23], gluteous fat [24] or inguinal region [5,25]. We preferred this location to others because it is easier to access, abundant quantities of fat, absence of surgical complications and production of a non visible scar.

Regarding cartilage healing, Murphy et al. published in 2003 that the use of MSCs deposited in a fibrin matrix would be useful [26]. However, although a recent equine study demonstrated early benefit, no significant differences were noted when MSCs plus fibrin were compared to fibrin alone at 8 months [27]. Based on that work, it is likely that modulation of the matrix or cells will need to be accomplished to observe long-term benefit of MSCs for cartilage repair. Treatment timing in relation to the degree of pathology could also be a factor contributing to the insignificant results of the equine study. Specifically, because MSCs appear to have a tropism for damaged cells, including fibrillated articular cartilage, it may be that at day 14 (day of treatment) the degree of fibrillation was not enough to allow an MSC treatment effect. However, more observation and studies on cases with more advanced fibrillation need to be conducted to answer this question. Following those results, our study was designed using animals with severe and chronic OA, where chondral degeneration and fibrillation were clearly present. These criteria could explain why our diseased dogs showed highly significant improvements in limb function.

Moreover, our study supports previously published data demonstrating that a single intraarticular administration of ADMSCs associated to PRGF-Endoret decreases pain and lameness in dogs with OA over at least a 6-month period [12], although our study was conducted in a different joint and our results were supported by objective kinetic data.

A follow up of six months could be considered as a standard for testing the evolution of a medical or surgical treatment, although our dogs always seemed to improve during the first month after treatment. Beyond this time lapse not significant changes were statistically detected. Recent clinical evaluation of this same treatment in a much larger group of animals with osteoarthritis in hip or/and elbow joints are showing that apparently improvement could be prolonged for about 10 month (JM Carrillo, unpublished observations) when dogs seemed to start to worsen. This is a fact that could be contrasted with a biomechanic evaluation of a homogeneous larger group of hip OA dogs of the same breed and should encourage researchers to objectively determine when a new cycle of treatment should be useful to stop a relapse.

Regarding statistical analysis, more complex models could have been considered but we chose this one because it offers an adequate compromise between complexity and ability to represent the relationships between the considered variables [28,29].

## Conclusion

Force platform analysis demonstrated quantitatively that ADMSC+ PRGF-Endoret therapy shows significant potential for clinical use in the treatment of lameness associated with OA.

Although this study was limited to a small number of dogs with severe OA, MSC therapy was found to be an appropriate treatment for hip joints, in terms of its efficacy in objectively improving the dogs’ gait and ability to live a more normal life, and the absence of side effects. In the future, ADMSCs could also become a promising therapeutic strategy in human OA.

## Methods

The research protocol was revised and authorized by the Ethical Committee of Animal Welfare (CEBA) of the University of Las Palmas de Gran Canaria (Spain) with reference code: 001/2010 CEBAULPGC.

### Animals

Eight adult client-owned Canarian Presa dogs (5 males, 3 females) with lameness and pain attributed to OA associated with hip dysplasia were included in the study. The dogs were affected by chronic OA and had not received any kind of medications (e.g., non-steroidal anti-inflammatory drugs, analgesics), nutraceuticals (e.g., glucosamine or chondroitin, vitamin E, omega-3 fish oil), or adjunctive therapies (e.g., acupuncture) for at least 2 months. A control group consisted of 5 sound and healthy dogs of the same breed.

None of the dogs were forced to perform physical activity. Dog owners were informed and granted a signed consent for the whole procedure.

Radiographs confirmed the presence of OA compatible with D and E degrees of hip dysplasia as defined by the Fédération Cynologique Internationale (World Canine Organization). D- degree dysplasic dogs showed obvious deviation from the normal with evidence of a shallow acetabulum, flattened femoral head, poor joint congruency, and in some cases, subluxation with marked changes of the femoral head and neck. E-degree dysplasic dogs showed complete dislocation of the hip and severe flattening of the acetabulum and femoral head [30]. Additional radiographs of knee and elbow joints were taken after physical and orthopedic examinations were performed to ensure that hip OA was the main reason for the observed clinical signs and that general health was otherwise normal.

### Extraction and culture-inoculation of ADMSCs and PRGF-Endoret

Stem cell extraction and inoculation phases were performed under general anesthesia (sevofluorane). Adipose tissue (20 g) was collected from the inguinal region through a small surgical incision and then included in a sterile bottle with culture medium for the cellular maintenance. Additionally, 120 ml of blood was extracted in sterilized conditions and deposited in 8 ml serum tubes. The adipose tissue and the blood tubes were sent to Fat-Stem Laboratories (Buggenhout, Belgium) where they were processed using the patent protocol of Dog Stem® (Fat-Stem). The number of messenchymal stem cells was over 30 million, shown in the laboratory certificate for the quality of the cells and were sent in two 2 ml tubes with 15 million per tube.

Once the AMSC were received they were infiltrated with the PRGF-Endoret that was prepared in that moment following a patented method [31]: 20 ml of blood were aseptically collected in four 4.5 ml citrate tubes, then centrifugated during 8 minutes at 460 G. Before the infiltration the PRGF was activated with 5% of its volume with 10% calcium chloride.

Subsequently, PRGF-Endoret was associated to ADMSC. The resultant 4 ml solution was injected aseptically into the hip joints through conventional arthrocentesis sites.

The needle was introduced just cranioproximal to the trochanter major, aimed slightly ventrally and caudally. The appearance of joint fluid confirmed proper needle placement [32].

### Gait analysis

Gait analysis was performed using a single platform mounted in the center of, and level with, a 7-m runway covered by a rubber mat. The mat weight was discarded setting to “0 force” with the tare button after the platform was covered. Dogs were leash guided at walk over the force platform by the same handler. Walk velocity was measured by use of a motion sensor (Pasco, California, USA) positioned 1 m from the platform.

Five valid trials, at a sampling frequency of 250 Hz, were obtained for each dog. A trial was considered valid when the limb fully contacted the force platform, and with the dog walking next to the handler without pulling on the leash. The trial was discarded if the dog was distracted during the measurement, if the limb struck the edge of the force plate, or if any portion of the contralateral paw hit the force plate. A member of the research team (BC) evaluated the trial to confirm which limb touched the center of the force platform.

The platform was interfaced with a dedicated computer using DataStudio (Pasco, California, USA), software specially designed for the acquisition, numerical conversion, and storage of data. A team member (JMV) recorded data from both affected limbs at day 0, 30, 90, and 180 post-treatment; the obtained PVF and VI values were normalized relative to body weight (%) to characterize the possible improvement of lameness during treatment with MSCs.

### Statistical analysis

Parameters in this model were estimated by using the package nlme in the R statistical software [33]. Data were analyzed by a different, blinded researcher (AS) who did not perform acquisition of data. For the analysis of these data, a linear mixed effects model for a blocked design with repeated measures was considered [28,29]. The experimental factor (time) and the status (lame-sound) of the dog were considered as fixed effects factors, while the blocking factor (dog) was a random effects factor. Because the dogs represent a random sample of the population of interest, any interaction terms modeling differences between dogs in its response when changing from different observation periods will also be expressed as random effects. Thus, the model we consider is of the form:

$$y_{\mathrm{ijkl}}=\beta_{i}+\gamma_{j}+{\left(\beta \gamma \right)}_{\mathrm{ij}}+b_{jk_{j}}+b_{\mathrm{ij}k_{j}}+\epsilon_{\mathrm{ij}k_{j}l}$$

with *i*=0,1,3,6 (months), *j*=0 (sound), 1 (lame), *k*_*0*_=1,…,8, *k*_*1*_=1,…,5, *l*=1,…,5. In this model, *β*_*i*_ represents the effect of time, γ_j_ the effect of the dog being sound or lame and (*βγ*)_ij_ the interaction between these factors. The term *b*_jk_represent the random effects of the dogs, and the *b*_ijk_ represents the random interaction terms between dogs and time, being:

$$b_{jk_{j}}∼N\left(0,\sigma_{1}^{2}\right),\;b_{\mathrm{ij}k_{j}}∼N\left(0,\sigma_{i}^{2}\right),\;\epsilon_{\mathrm{ij}k_{j}l}∼N\left(0,\sigma^{2}\right)$$

Significance of the differences in PVF and VI between periods of observation were tested by means of analysis of variance of these models. Following this analysis, post-hoc comparisons between fixed effects were performed using Tukey’s procedure. For assessing the validity of the model, the Shapiro-Wilk test was applied for testing normality of the residuals.

For assessing the relationships between supporting force in the more-lame and the less-lame limbs and also between vertical impulses in the two limbs, a regression model with random effects of dog on slope and intercept was used:

$$y_{\mathrm{ij}}=\beta_{0}+b_{0}+\left(\beta_{1}+b_{1}\right)x_{i}+\epsilon_{\mathrm{ij}}$$

with:

$$b_{0}∼N\left(0,\sigma_{0}^{2}\right),\;b_{1}∼N\left(0,\sigma_{1}^{2}\right),\;\epsilon_{\mathrm{ij}}∼N\left(0,\sigma^{2}\right)$$

Here y_ij_ represents the value (PVF or VI) in the less-lame limb and x_ij_ the value in the more-lame limb.

Significance level was set at *P* ≤ 0.05 in all tests.

## Abbreviations

ADMSCs: Adipose-derived mesenchymal stem cells; MSCs: Mesenchymal stem cells; OA: Osteoarthritis; PP: Platelet-poor plasma; PRGF: Plasma rich in growth factors; PVF: Peak vertical force; VI: Vertical impulse; N: Newton.

## Competing interests

The authors declare that they have no competing interests.

## Authors’ contributions

JMV, MR and JMC designed the study, drafted the manuscript, and analyzed data; GS and RC revised and edited the manuscript; AS designed and developed the statistical analysis; MM and BC performed the selection of animals and helped with the revision of the manuscript.JMV and AS were blind researchers. All authors read and approved the final manuscript.
